# Facile Pretreatment of Three-Dimensional Graphene through Electrochemical Polarization for Improved Electrocatalytic Performance and Simultaneous Electrochemical Detection of Catechol and Hydroquinone

**DOI:** 10.3390/nano12010065

**Published:** 2021-12-27

**Authors:** Huaxu Zhou, Guotao Dong, Ajabkhan Sailjoi, Jiyang Liu

**Affiliations:** 1 Key Laboratory of Surface & Interface Science of Polymer Materials of Zhejiang Province, Department of Chemistry, Zhejiang Sci-Tech University, Hangzhou 310018, China; chemist1996@126.com (H.Z.); ajabkhan@163.com (A.S.); 2 Heihe Water Resources and Ecological Protection Research Center, Lanzhou 730030, China

**Keywords:** three-dimensional graphene, electrochemical polarization, electrocatalytic, individual and selective determination, isomers of benzenediol

## Abstract

Three-dimensional graphene (3DG) with macroporous structure has great potential in the field of electroanalysis owing to a large active area, excellent electron mobility and good mass transfer. However, simple and low-cost preparation of 3DG electrodes with high electrocatalytic ability is still a challenge. Here, a fast and convenient electrochemical polarization method is established to pretreat free-standing 3DG (p-3DG) to offer high electrocatalytic ability. 3DG with monolithic and macroporous structure prepared by chemical vapor deposition (CVD) is applied as the starting electrode. Electrochemical polarization is performed using electrochemical oxidation (anodization) at high potential (+6 V) followed with electrochemical reduction (cathodization) at low potential (−1 V), leading to exposure of edge of graphene and introduction of oxygen-containing groups. The as-prepared p-3DG displays increased hydrophilicity and improved electrocatalytic ability. As a proof of concept, p-3DG was used to selective electrochemical detection of two isomers of benzenediol, hydroquinone (p-BD) and catechol (o-BD). In comparison with initial 3DG, p-3DG exhibits increased reversibility of redox reaction, improved peak current and good potential resolution with high potential separation between p-BD and o-BD. Individual or selective determination of p-BD or o-BD in single substance solution or binary mixed solution is realized. Real analysis of pond water is also achieved.

## 1. Introduction

Graphene, atomically thin nanocarbon with sp^2^-bonded carbon packed into a two-dimensional (2D) honeycomb lattice, is one of the most popular research topics in the fields of chemistry, materials, and physical science [1,2]. Graphene is considered as an ideal electrode material for electrochemical sensing owing to intriguing physicochemical properties including large theoretical specific surface area (2630 m^2^g^−1^), extraordinary electronic transport property, facile (bio)functionalization and good biocompatibility [3,4]. However, strong π-π interaction between graphene sheets leads to inevitable aggregation and restacking, which significantly hinder the performance of graphene by reducing the surface area or active sites, and decreasing mass transfer [5,6]. At the same time, the electrocatalytic performance of graphene is strongly influenced by the surface chemistry. Therefore, facile tailoring the morphology and surface chemistry of graphene to avoid aggregation, increase surface area, and offer high electrocatalytic performance is highly desirable.

Integrating graphene nanosheets into a macroscopic three-dimensional (3D) structure can effectively solve the problem of repacking and agglomeration. The 3D graphene materials, which are built from unstacked 2D graphene sheets, not only inherit the outstanding properties of 2D graphene nanosheets, but also offer unhindered diffusion of substances, large electroactive areas, and improved structural stability [7,8,9]. Unlike solution-phase self-assembly method (e.g., assembly of chemically exfoliated graphene oxide sheets followed by reduction), chemical vapor deposition (CVD) facilitates facile and scalable production of high-quality 3D graphene with controllable crystallinity and layer numbers. For instance, 3D graphene foam (3DG) synthesized using nickel foam templated CVD process has been one of the main forms of 3D graphene materials and been successfully commercialized [10,11]. The 3DG is a monolith with continuous and seamlessly interconnected graphene network, which provides a unique and stable macroporous scaffold with high conductivity and offers great potential for the construction of functional 3D electrodes. However, high hydrophobicity and defect-free structure of 3DG compromise the electrocatalytic performance. The establishment of a simple and universal method to improve the hydrophilicity and electrocatalytic activity of 3DG can greatly expand its application in the field of electroanalysis.

At molecular level, graphene nanosheets have two different surfaces, namely the base surface and the edge site. The basal plane represents the sp^2^-bonded carbon atoms perfectly arranged in the honeycomb lattice, while the edge is an atom-thick line of carbon atoms with dangling bonds or other capping groups [12,13,14]. In order to improve the electrocatalytic activity of graphene, it is necessary to expose the edges of graphene and introduce active groups. For instance, oxygen-containing groups offer active centers for redox system on electrode surface and facilitate reaction kinetics, leading to significant improvement of sensitivity towards target analytes. In addition, the introduced oxygen-containing groups also improve the hydrophilicity of graphene, increase electronic density of states and affect electric double layer. Therefore, the exposure of basal planes and introduction of oxygen-containing groups into graphene materials can effectively improve the hydrophilicity and electrocatalytic activity of graphene.

Electrochemical polarization has been proven to be a simple and effective pretreatment strategy that can remarkably improve the sensitivity and selectivity of carbonaceous electrode [15,16,17,18]. Generally, electrochemical polarization includes anodic polarization under positive voltage (electrochemical oxidation) and cathodic polarization under negative voltage (electrochemical reduction). It has been proven that during the anodic polarization process, carbon electrode is oxidized and various oxygen-containing groups such as hydroxyl, carboxyl, ketone, etc. appear on the surface. For graphene electrodes, anodic polarization will further promote the exposure of new edge planes. In the subsequent cathodic polarization, the electrochemical reduction process will reduce part of oxygen-containing groups (e.g., reducing carbonyl group to hydroxyl group) and restore the conductivity of the electrode. Thus, electrochemical polarization substantially endows carbon electrode with improved sensitivity and selectivity towards target analytes by generating active sites and functional groups, that facilitate electron transfer and improve wettability. Compared with complex modification processes, electrochemical polarization has the advantages of simplicity, sensitivity, high efficiency, low cost, and environmental friendliness.

In this work, we demonstrate an electroanalysis platform to distinguish electroactive isomer based on electrochemical polarization of three-dimensional graphene (p-3DG). The simple electrochemical polarization offers p-3DG with abundant edge active sites and functional groups, leading to high hydrophilicity and excellent electrocatalytic activity. Combined with the favorable mass transport and high active area of macroporous 3D electrode, p-3DG exhibits two couples of independent well-defined redox peaks and wide peak-to-peak separation toward hydroquinone (p-BD) and catechol (o-BD) as the proof-of-concept demonstrations. Owing to the facile and efficient functionalization process, p-3DG stands for a new type of functional material for the fabrication of free-standing electrochemical sensors with high sensitivity and good potential discrimination ability. 

## 2. Materials and Methods

### 2.1. Chemicals and Materials

Disodium hydrogen phosphate (Na_2_HPO_4_•7H_2_O), sodium dihydrogen phosphate (Na_2_H_2_PO_4_), catechol (o-BD), and 1-Butyl-3-methylimidazolium hexafluorophosphate (BMIMPF_6_) were purchased from Aladdin (Shanghai, China). Hydrochloric acid (HCl) was purchased from Hangzhou Shuanglin Chemical Engineering Co., Ltd. (Hangzhou, China). Hydroquinone (p-BD) and acetonitrile (99.9%) were purchased from Macklin (Shanghai, China). Electronic glass substrate/soda lime glass was purchased from Zhuhai Kaivo Optoelectronic Technology Co., Ltd. (Zhuhai, China). Pond water was from Zhejiang Sci-Tech University (Hangzhou, China). All chemicals and reagents were of analytical grade and used as received without further purification. Ultrapure water (18.2 MΩ·cm) was used to prepare all aqueous solutions in this work.

### 2.2. Measurements and Instrumentations

Scanning electron microscopy (SEM) investigation was performed on a SU8100 microscope (Hitachi Ltd., Tokyo, Japan) at an acceleration voltage of 10 kV. The Raman spectrum was recorded using the CRM200 Raman system (WITeck, Ulm, Germany) when excited using 514 nm laser. X-ray photoelectron spectroscopy (XPS) was obtained with Mg Ká radiation (Perkin Elmer, Waltham, MA, USA). All electrochemical measurements, including cyclic voltammetry (CV) and differential pulse voltammetry (DPV) were performed on the Autolab PGSTAT302N electrochemical workstation (Metrohm, Herisau, Switzerland). A conventional three electrodes system was employed. Briefly, a 3DG or p-3DG was applied as the working electrode and platinum wire was used as the counter electrode. The reference electrode was an Ag/AgCl electrode (saturated with KCl). For DPV measurement, the experimental parameters used were as follows: step, 0.005 V; modulation amplitude, 0.025 V; modulation time, 0.05 s; interval time, 0.2 s.

### 2.3. Preparation of 3DG Electrode

According to a previous report [11], 3DG was synthesized using chemical vapor deposition (CVD) with foamed nickel as the growth substrate. The Ni foam was then removed through incubation in HCl solution (3 M) at 80 °C for 24 h. To prepare the 3DG electrode, a 3DG foam (0.5 cm × 0.5 cm) was fixed on a glass slide and connected with copper wire using conductive silver glue. Then, the silver glue and copper wire were sealed with silica gel for insulation.

### 2.4. Electrochemical Polarization of 3DG Electrode to Prepare p-3DG

3DG electrode was electrochemically polarized using both anodic oxidation and cathodic reduction. Ionic liquid 1-butyl-3-methylimidazole ammonium hexafluorophosphate (BMIMPF_6_) in acetonitrile (20%, *v*/*v*) was applied as the electrolyte for anodic oxidation. Briefly, a potential of + 6V was applied on 3DG for 80 s. Subsequently, the obtained electrode was applied at a voltage of −1.0 V in a phosphate buffer solution (0.1 M PBS, pH 6.5) for cathodic reduction. The resulting electrode was abbreviated as p-3DG.

### 2.5. Electrochemical Detection of p-BD and o-BD

Phosphate buffer solution (PBS, 0.1 M, pH 7) was chosen as the electrochemical electrolyte for the detection of p-BD or o-BD. Briefly, the electrochemical responses of p-BD and o-BD on p-3DG were measured individually or simultaneously by CV or DPV. To assess the practical application of the proposed p-3DG sensor, the standard addition method was applied in detection of p-BD and o-BD in real environmental sample-pond water. After pond water was filtered through a 0.22 μm film and diluted using PBS by a factor of 10, various concentrations of p-BD or o-BD were spiked and detected.

## 3. Results and Discussion

### 3.1. Electrochemical Polarization of Three-Dimensional Graphene

**Figure 1:**
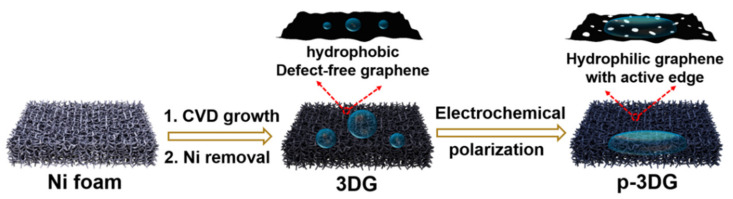
Schematic illustration for the preparation of p-3DG through electrochemical polarization of CVD-grown 3DG.

Three-dimensional graphene (3DG) has defect-free graphene and monolithic structure with macroporous structure. The unique structure can avoid the agglomeration of 2D graphene while maintaining the high electron transfer of graphene and ensuring the high mass transfer of substrates [19]. However, high hydrophobicity and low electrocatalytic property limit the wide application of 3DG in electroanalysis. Figure 1 illustrates the preparation of pretreated 3DG (p-3DG) by electrochemical polarization. Anodization is firstly carried out at a high potential (+6 V) followed by electrochemical reduction at low potential (−1 V). In order to avoid a large amount of gas generated by the decomposition of water during anodization to destroy the structure of the three-dimensional graphene, we choose ionic liquid diluted in acetonitrile as the supporting electrolyte. Then, the electrolyte was changed to weakly acidic PBS solution in the subsequent cathodization to introduce oxygen-containing groups. 

**Figure 2:**
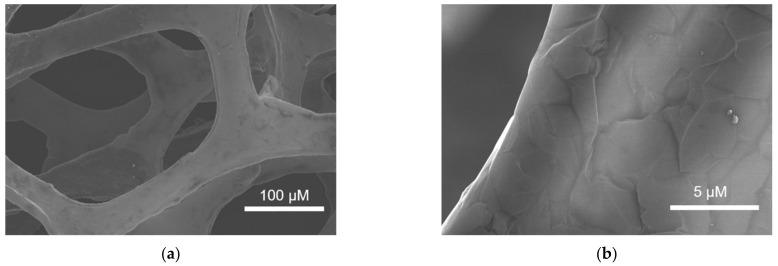

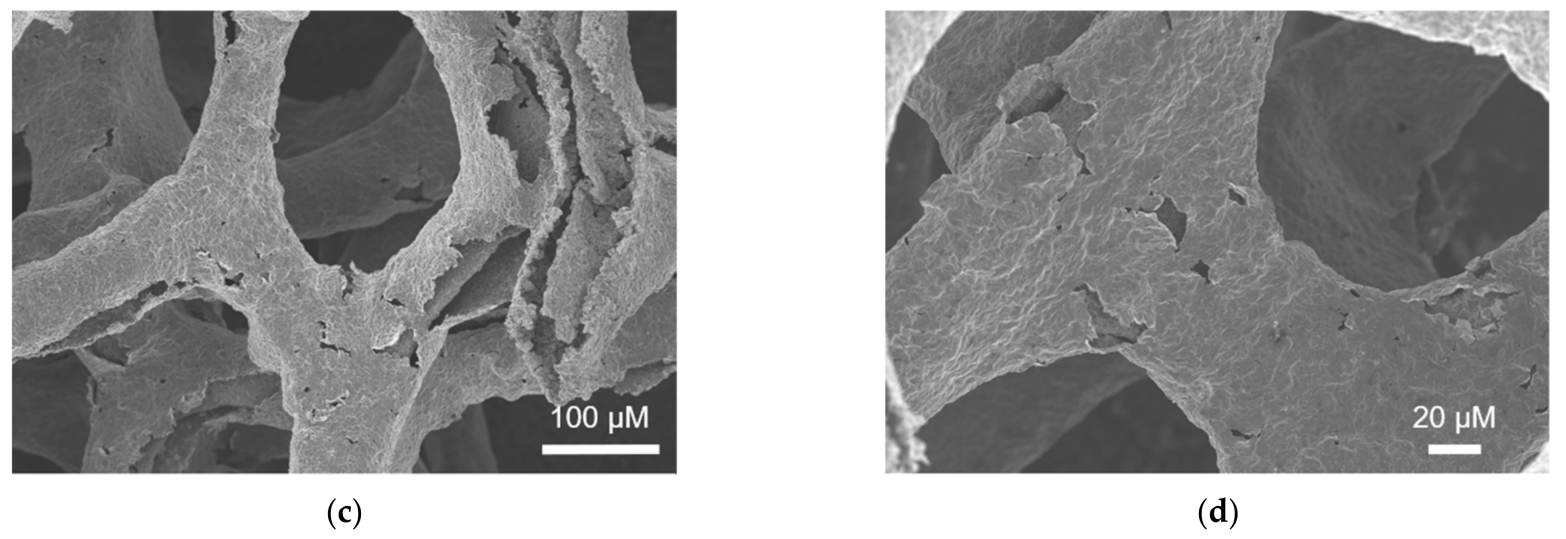
SEM images of 3DG (**a**,**b**) and p-3DG (**c**,**d**) at different magnificence.

Figure 2a,b shows typical SEM images of 3DG at different magnifications. It can be seen that 3DG has a monolithic structure with macroporous structure and smooth graphene structure. After electrochemical polarization, p-3DG still maintains a three-dimensional macroporous structure, but some cracks are generated on the surface, which facilitates the exposure of the edge of graphene.

### 3.2. Structure and Composition Characterization of p-3DG

**Figure 3:**
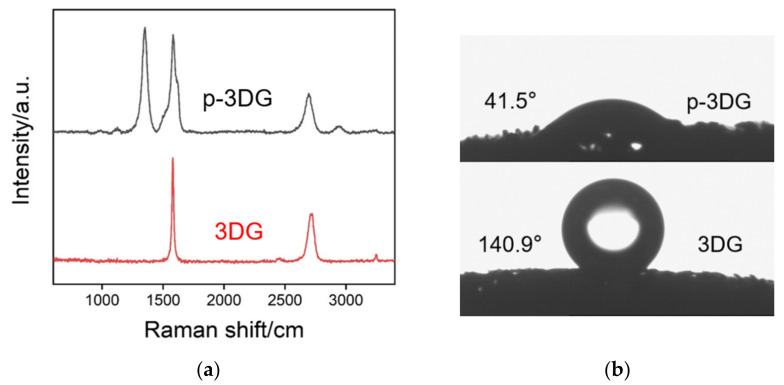

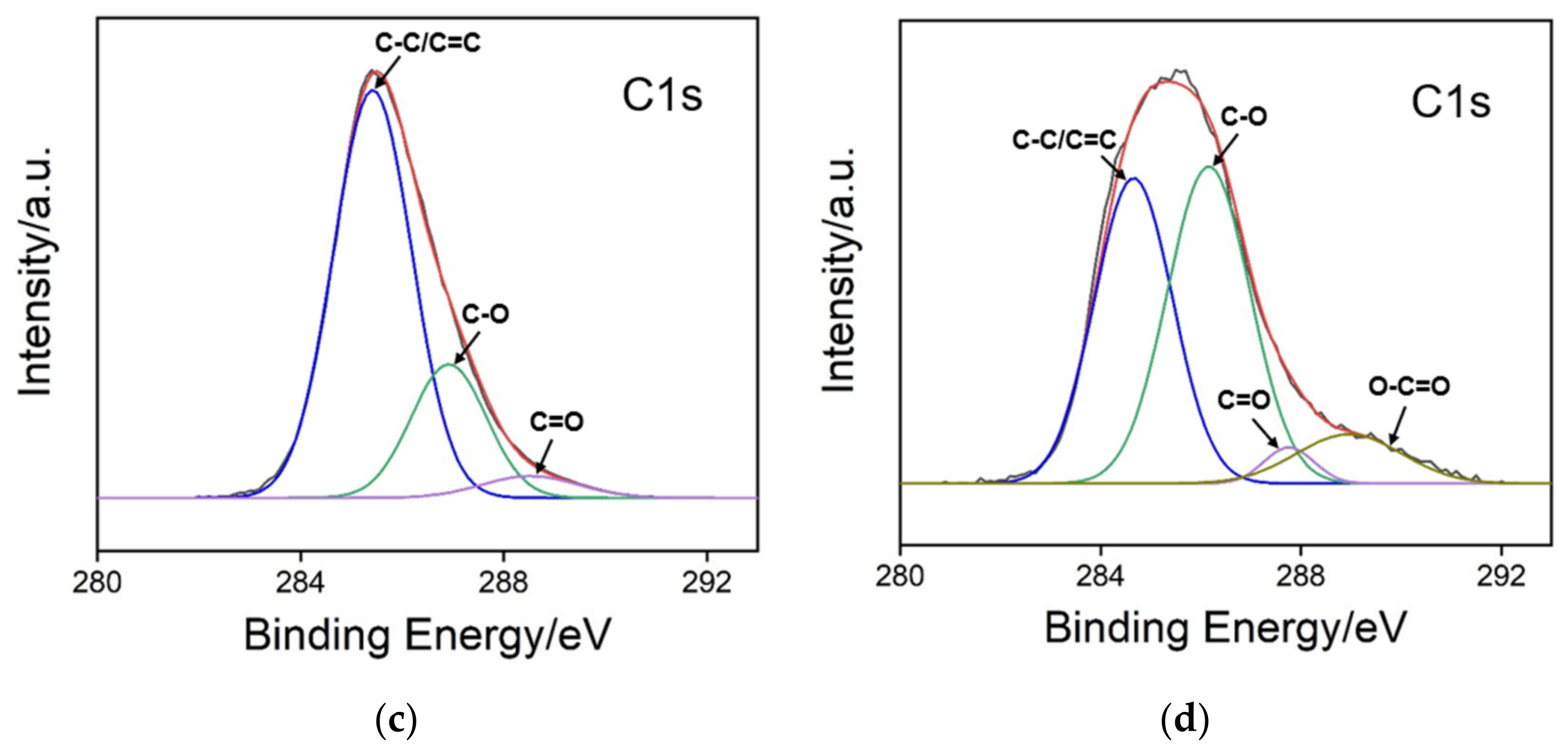
Raman spectra (**a**) and contact angle images (**b**) of 3DG and p-3DG. High-resolution X-ray photoelectron spectrum (XPS) of C1s peaks from 3DG (**c**) and p-3DG (**d**).

Raman spectroscopy is employed to investigate the structure change of 3DG in the electrochemical polarization. Three characteristics of graphene, including D band, G band, and 2D band were investigated. Generally, G band represents the E_2g_ phonon of sp^2^ bonds of carbon atoms, while D band reflects the defect structure with sp^3^-hybridized carbon [20,21,22]. In case of a 2D band, it is the two-phonon band that is activated by the double resonance at the zone boundary. For the initial 3DG, two main peaks are observed that corresponds to the D band and 2D bands (Figure 3a). When electrochemical polarization was performed, a new D band appeared on p-3DG in addition to the G band and 2D band, indicating surface defects with sp^3^-hybridized carbon. Thus, the simple electrochemical polarization creates defects and promotes the exposure of the edge sites of graphene and introduction of functional groups. In addition, the hydrophobicity of 3DG or p-3DG was also investigated. As shown in Figure 3b, 3DG exhibits high hydrophobicity with a contact angle of 140.9°, indicating low wettability in aqueous solution. On the contrary, p-3DG displays a contact angle of 41.5°, suggesting good hydrophilicity. Thus, the strong hydrophobicity, one drawback of 3DG, can be overcome by simple electrochemical polarization. 

The changes of chemical groups on the surfaces of the two electrodes before and after electrochemical polarization can be further characterized by X-ray photoelectron spectroscopy (XPS). Figure S1 (in Supporting Information, SI) shows the XPS survey spectrum of 3DG. No Ni 2p peak (~875–850 eV) was observed, indicating that there was no residual Ni in 3DG after removing the Ni template with hot HCl aqueous solution. Figure 3c,d give the high-resolution XPS of C1s peaks from 3DG and p-3DG. For 3DG, the high content of C–C/C = C proves the sp^2^-bonded carbon of graphene. The small signals of oxygen-related groups might result from the chemical bonding of O_2_ or H_2_O on graphene in the atmosphere. For p-3DG, the decrease of sp^2^-bonded carbon and the increase of oxygen-containing groups also prove the introduction of abundant defects and functional groups.

### 3.3. Electrocatalytic Activity of p-3DG towards Hydroquinone and Catechol

Hydroquinone (1,4-benzenedihydroxy, p-BD) and catechol (also 1,2-benzenedihydroxy, o-BD) are two important isomers of benzenediol, which are widely used in many industries (e.g., dyes, plasticizers, cosmetics, pesticides, etc.) and distribute in environments [23,24,25,26]. Owing to high toxicity and difficulty in degradation, p-BD and o-BD have become important pollutants and co-existed in environmental samples. Due to the similar structures and properties, simultaneous determination of p-BD and o-BD is of great significance [26,27]. In comparison with other methods (e.g., fluorescent [28,29]), the electrochemical sensor has the advantages of sensitive detection, convenient operation, and simple instrumentation. However, the detection potentials of catechol and hydroquinone are close, which means that conventional electrodes cannot achieve simultaneous determination of p-BD and o-BD. Thus, developing a simple and efficient strategy to improve the electrocatalytic performance of the electrode to realize simultaneous detection of p-BD and o-BD is highly desirable.

**Figure 4:**
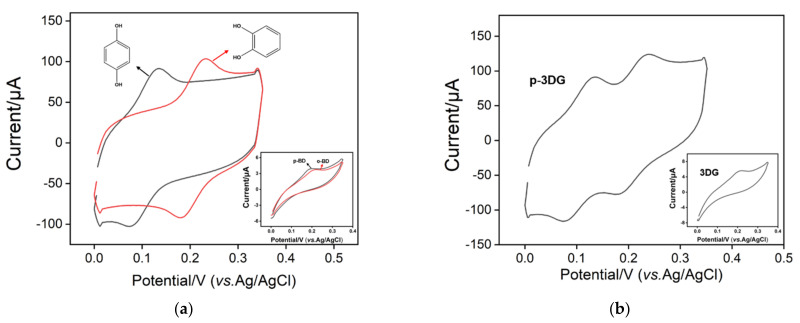
(**a**) Cyclic voltammetry curves of p-BD (50 μM) or o-BD (50 μM) on p-3DG or 3DG (inset). (**b**) Cyclic voltammetry curve of mixture of p-BD and o-BD (both 50 μM) at p-3DG or 3DG (inset) electrode. The electrochemical electrolyte is 0.1 M PBS solution (pH 7). The scan rate was 100 mV/s.

As shown in the inset of Figure 4a, p-BD and o-BD show irreversible electrochemical oxidation-reduction processes on 3DG electrodes. In addition, the corresponding peak potentials for electrochemical oxidation are very close, indicating that p-BD and o-BD cannot be electrochemically distinguished. When 3DG electrodes are used to analyze a mixture of p-BD and o-BD, only one peak appears (inset of Figure 4b). On the contrary, both p-BD and o-BD exhibit reversible electrochemical oxidation and reduction peaks on p-3DG (Figure 4a). In comparison with the oxidation peak potential of o-BD (0.12 V), the oxidation peak potential of p-BD is 0.23 V. A separation of the peak potential is greater than 110 mV, indicating potential for simultaneous detection of p-BD and o-BD. In addition, compared with the 3DG electrode, p-3DG exhibits a higher charging current, which indicates that the electrochemical polarization process increases the hydrophilicity of the electrode. Even when p-BD and o-BD are analyzed on p-3DG, distinguishable electrochemical oxidation and reduction peaks are detected (Figure 4a). The improved electrocatalytic performance comes from the exposure of graphene edge and the introduction of oxygen-containing groups caused by electrochemical polarization. These defects and functional groups can be used as active sites on the electrode surface to facilitate redox reactions by exchanging protons or electrons and to improve the hydrophilicity, leading to high electrocatalytic ability with good potential resolution. CV characterizations of 3DG and p-3DG electrodes in 0.1 M PBS (pH 7.0) were carried out to investigate whether there was Ni residue in the electrodes (Figure S2 in SI). As shown, no redox signals of Ni (~−0.2 V) are observed on both 3DG and p-3DG even though the p-3DG has high charging current signal resulting from the increase of active electrode area through electrochemical polarization. This result further proves that there was no residual nickel template in the prepared 3DG electrode. In comparison with modification strategies using complex modification processes and materials to improve electrocatalytic ability, our electrochemical polarization is simple and efficient [30,31].

### 3.4. Electrocatalytic Activity of p-3DG towards Hydroquinone and Catechol

The influence of pH value on electrochemical signal was investigated. As shown in Figure S3a (SI), p-BD has the highest electrochemical oxidation peak current at pH 7. Figure S3b is the linear regression line of electrochemical oxidation peak potential and pH. As seen, the increase of pH leads to the negative shift of the peak potential. In addition, a good linear relationship between potential and pH is revealed with a slope of 63 mV/pH, that is close to 59 mV/pH, indicating that p-BD undergoes an oxidation-reduction reaction on electrode with the same number of protons and electrons. In case of o-BD, the highest peak current is also observed when the pH is 7 and the peak potential shifts to negative potential with the increase of pH (Figure S3c,d). The potential and pH have a good linear relationship with a slope of 59 mV/pH, suggesting the same number of protons and electrons in oxidation-reduction reaction. 

**Figure 5:**
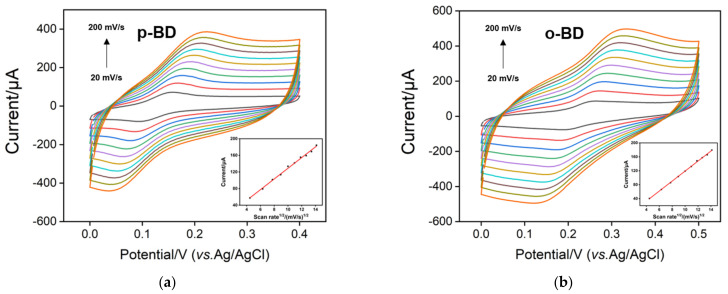
Cyclic voltammetry curves of p-BD (**a**) and o-BD (**b**) on p-3DG at different scan rates. The insets show the dependence of anodic peak currents on the square root of scan rate.

Figure 5 shows the cyclic voltammogram curves of p-BD and o-BD on p-3DG electrode under different scan numbers. As the scan rate increases, both the oxidation and reduction peak currents increase. As shown in the insets in Figure 5, the oxidation peak current is proportional to the square root of the sweep speed, indicating that the electrochemical redox of both p-BD and o-BD are diffusion controlled.

### 3.5. Individual and Selective Detection of p-BD and o-BD

**Figure 6:**
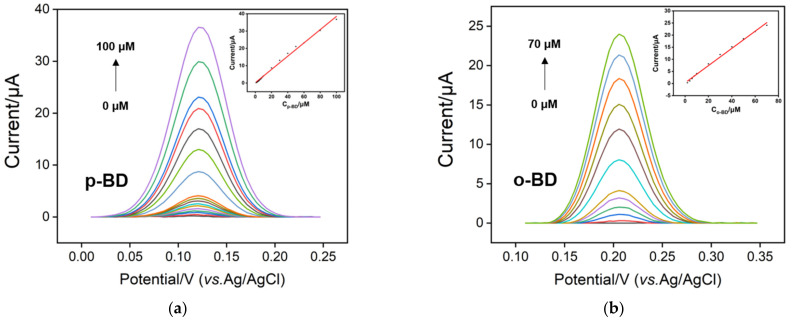
DPV responses of p-3DG electrode to various concentrations of p-BD (**a**) or o-BD (**b**) in 0.1 M PBS (pH 7). The insets show the corresponding calibration curves. Error bars denote the standard deviations of three measurements.

**Figure 7:**
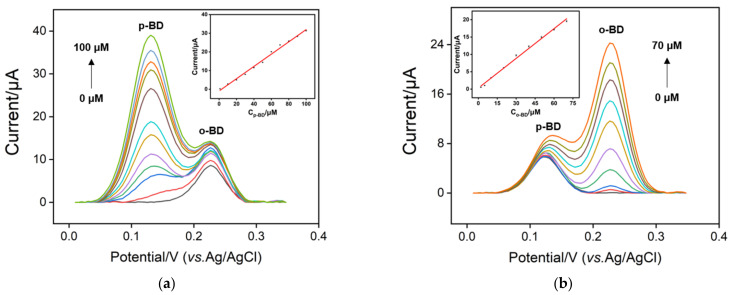
(**a**) DPV responses of p-3DG electrode to various concentrations of p-BD in presence of o-BD (20 μM). (**b**) DPV responses of p-3DG electrode to various concentrations of o-BD in presence of p-BD (20 μM).

Figure 6 displays differential pulse voltammetry (DPV) responses obtained on p-3DG electrode to various concentrations of p-BD or o-BD in PBS (0.1 M, pH 7). The insets show the corresponding calibration curves. The oxidation peak current is linear proportional to the concentration of p-BD in the range of 1 μM to 100 μM (I = 0.383 C + 0.211, R^2^ = 0.994). A limit of detection (LOD) is calculated as 40 nM at a signal-to-noise ratio of 3. For o-BD, the p-3DG sensor is able to detect o-BD with a linear range of 2–70 μM (I = 0.355 C + 0.307, R^2^ = 0.994) and LOD of 0.12 μM. Selective detection of o-BD or p-BD in the binary mixture is also investigated. As shown in Figure 7, the DPV oxidation peak current is proportional to the concentration of p-BD from 1 μM to100 μM (I = 0.330 C-0.908, R^2^ = 0.992) in presence of o-BD (20 μM). For the detection of o-BD in presence of p-BD (20 μM), the DPV oxidation peak current is linearly proportional to the concentration of o-BD from 2 μM to 70 μM (I = 0.285 C + 0.313, R^2^ = 0.994) with an LOD of 0.21 μM. It is worth noting that the selective detection of p-BD or o-BD in the binary mixture shows a similar detection linear range and sensitivity as the individual detection, indicating the possibility of simultaneous detection.

### 3.6. Real Sample Analysis

In order to verify the feasibility of p-3DG electrode in practical applications, the determination of p-BD or o-BD in presence of the other isomers in the pond lake is also investigated (Table 1 and Table 2). As shown, the standard addition method was employed to determine the artificial concentrations of p-BD or o-BD by spiking a certain amount of analyte into the pond lake samples. The recovery rate was between 95.2–103.3% and the relative standard deviation (RSD) is no more than 3.1%, indicating its application potential in real analysis.

## 4. Conclusions

In summary, we have developed a simple electrochemical polarization strategy to pretreat three-dimensional graphene (3DG) electrodes and expanded its application as an electrochemical sensing platform. The pretreated 3DG (p-3DG) shows remarkable electrocatalytic performance. Taking the pair of isomers of hydroquinone (p-BD) and catechol (o-BD) as an example, p-3DG improves the reversibility of the electrochemical redox process, increases the peak current, and has a significant potential distinguishing ability in comparison with the initial 3DG electrode. Since 3DG prepared by chemical vapor deposition (CVD) has now been commercially produced and sold, the effective electrochemical polarization strategy established here can open up new ways for the functionalization of three-dimensional graphene. In addition, p-3DG has great potential in electrochemical sensing, energy storage and electrocatalysis.

## Figures and Tables

**Table 1:** Determination of p-BD in pond water sample in the presence of o-BD.

Sample	Added p-BD (μM)	Added o-BD (μM)	p-BD Found (μM)	RSD (%)	Recovery (%)
Pond water	5	20	5.1	3.0	102.0
	15	20	14.3	1.6	95.2
	50	20	49.6	0.3	99.2

**Table 2:** Determination of o-BD in pond water sample in the presence of p-BD.

Sample	Added o-BD (μM)	Added p-BD (μM)	o-BD Found (μM)	RSD (%)	Recovery (%)
Pond water	5	20	4.8	2.5	96.0
	15	20	15.5	3.1	103.3
	50	20	51.2	1.9	102.4

## Data Availability

The data presented in this study are available on request from the corresponding author.

## Supplementary Materials

The following are available online at https://www.mdpi.com/article/10.3390/nano12010065/s1, Figure S1: X-ray photoelectron spectroscopy characterization of p-3DG electrode. Figure S2: Cyclic voltammetry characterizations of different electrodes in buffer solution. Figure S3: The influence of pH value on electrochemical signal.
