# Supplementary material for: Facile Pretreatment of Three-Dimensional Graphene through Electrochemical Polarization for Improved Electrocatalytic Performance and Simultaneous Electrochemical Detection of Catechol and Hydroquinone
Source: Nanomaterials (Basel). 2021 Dec 27;12(1):65. doi: 10.3390/nano12010065 (PMC8746492; doi:10.3390/nano12010065)
Supplement: Supplementary file 1 [file nanomaterials-12-00065-s001.zip › nanomaterials-1510946-supplementary.pdf]

nanomaterials

Supplementary Materials

Facile Pretreatment of Three-Dimensional Graphene through Electrochemical Polarization for Improved Electrocatalytic Performance and Simultaneous Electrochemical Detection of Catechol and Hydroquinone

Huaxu Zhou [1,2], Guotao Dong [2,*], Ajabkhan Sailjoi [1] and Jiyang Liu [1,*]

1 Key Laboratory of Surface & Interface Science of Polymer Materials of Zhejiang Province, Department of Chemistry, Zhejiang Sci-Tech University, Hangzhou 310018, China; chemist1996@126.com (H.Z.); ajabkhan@163.com (A.S.)
2 Heihe Water Resources and Ecological Protection Research Center, Lanzhou, 730030, China
* Correspondence: dongguotao@hhglj.yrcc.gov.cn (G.D.); liujyxx@126.com (J.L.)

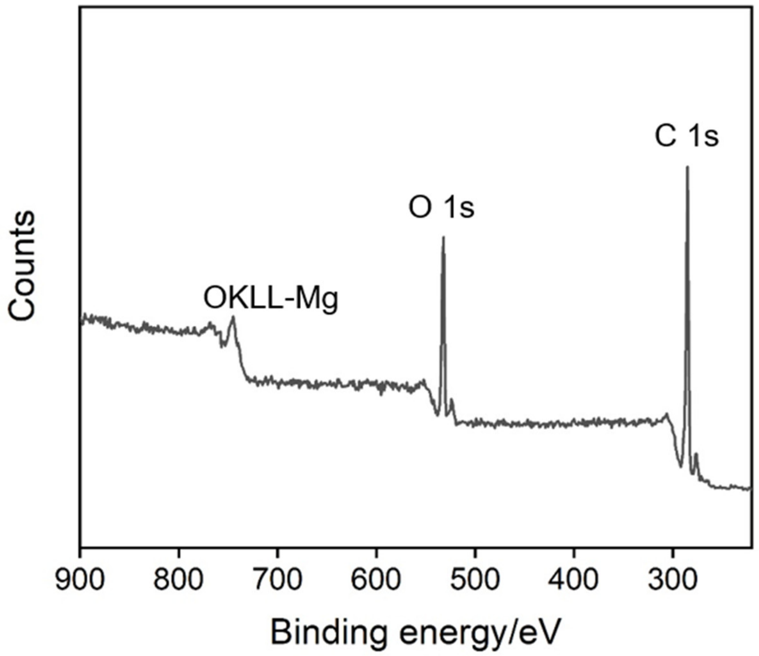

Figure S1. XPS survey spectrum of 3DG.

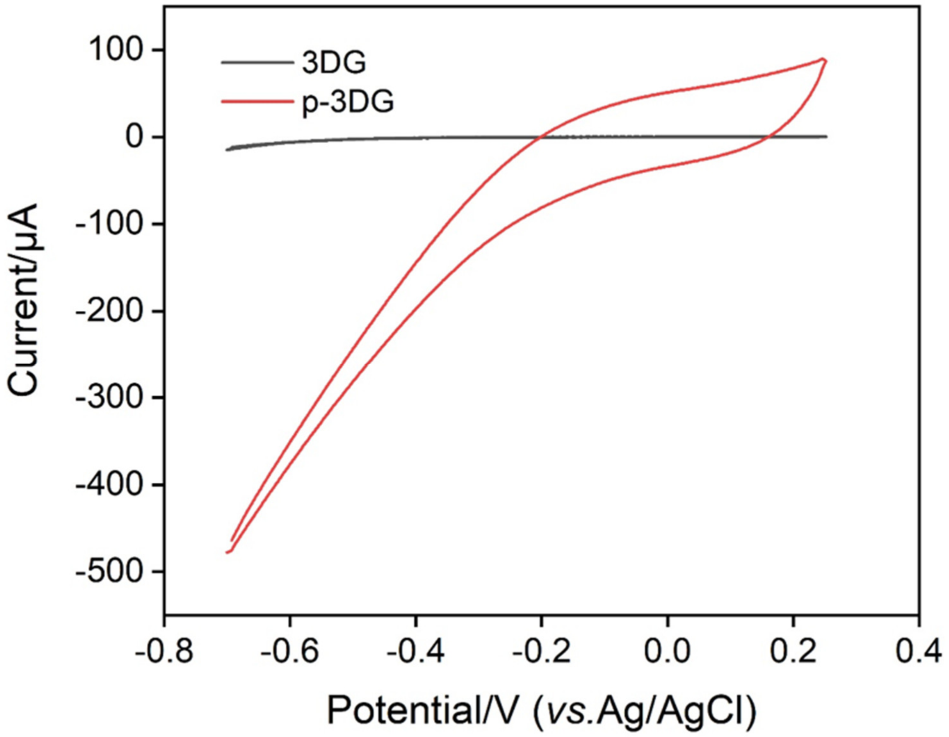

Figure S2. Cyclic voltammetry curves obtained on p-3DG or 3DG in 0.1 M PBS solution (pH 7.0). The scan rate was 100 mV/s.

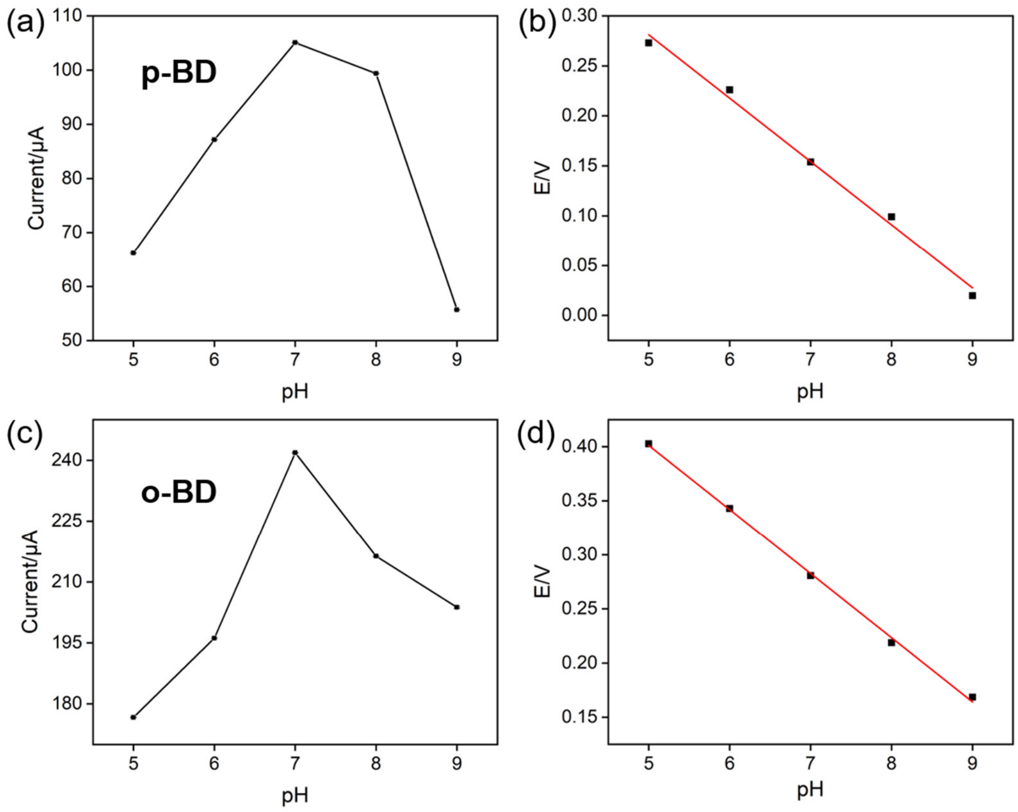

Figure S3. The electrochemical oxidation peaks of p-BD (a) or o-BD (c) at different pH. The linear regression line between anodic peak potential of p-BD (b) or o-BD (d) with pH values.
